# Surveillance for Antimicrobial Resistance in Gonorrhea: The Alberta Model, 2012–2016

**DOI:** 10.3390/antibiotics7030063

**Published:** 2018-07-20

**Authors:** Jennifer Gratrix, Anmmd Kamruzzaman, Irene Martin, Petra Smyczek, Ron Read, Lindsay Bertholet, Prenilla Naidu, Ameeta E. Singh

**Affiliations:** 1 STI Centralized Services, Population, Public and Indigenous Health, Alberta Health Services, Edmonton, AB T5J 3E4, Canada; jennifer.gratrix@ahs.ca (J.G.); petra.smyczek@ahs.ca (P.S.); lindsay.bertholet@ahs.ca (L.B.); 2 Surveillance and Reporting, Population, Public and Indigenous Health, Alberta Health Services, Calgary, AB T2W 3N2, Canada; anmmd.kamruzzaman@ahs.ca; 3 National Microbiology Laboratory, Winnipeg, MB R3E 3R2, Canada; irene.martin@canada.ca; 4 Department of Medicine, University of Calgary, Calgary, AB T2N 1N4, Canada; ron.read@ahs.ca; 5 Department of Medical Microbiology and Immunology, University of Alberta, Edmonton, AB T6G 3E1, Canada; prenilla.naidu2@ahs.ca; 6 Department of Medicine, University of Alberta, Edmonton, AB T6G 2G3, Canada

**Keywords:** gonorrhea, antimicrobial resistance, surveillance, sexually transmitted infections

## Abstract

Alberta established a surveillance system in 2001 to monitor resistance to antibiotics used for the treatment of gonorrhea. A retrospective review of gonorrhea cases during the last five years was conducted. All cases of gonorrhea were reportable to public health by testing laboratories and clinicians. Specimens were primarily submitted for nucleic acid amplification testing (NAAT); three sentinel sites obtained specimens for culture and NAAT. The Provincial Laboratory for Public Health conducted E-tests on isolates for multiple antibiotics. A proportion of isolates and NAAT specimens were submitted to the National Microbiology Laboratory for sequence typing (ST). Data were combined and analyzed using SAS version 9.4. Between 2012 and 2016, 13,132 gonorrhea cases were reported; 22.0% (*n* = 2891) had isolates available for susceptibility testing. All culture positive isolates were susceptible to ceftriaxone. Decreased susceptibility (0.5 ug/mL) to cefixime was reported in four cases in 2014. Resistance to azithromycin (≥2 ug/mL) ranged between 0.4% and 1.8%. Many (*n* = 509) unique STs were identified; the most prevalent sequence groups (SG) were SG-7638 (*n* = 367), SG-5985 (*n* = 145), and SG-11299 (*n* = 127). The Alberta model for maintaining surveillance for antimicrobial resistance in gonorrhea employs culture and NAAT specimens, providing information crucial to informing provincial treatment guidelines.

## 1. Introduction

*Neisseria gonorrhoeae* (NG) is a common, global sexually transmitted infection (STI) and the second most commonly reported STI in Canada [1]. There has been a gradual and steady increase in reported cases of gonococcal infections in Canada since 1997 [1]. Untreated infections can lead to pelvic inflammatory disease, infertility, and ectopic pregnancy in females and epididymitis and infertility in males [2]. Similar to other STIs, gonorrhea can also increase the risk of acquisition and transmission of human immunodeficiency virus (HIV) [3].

Over time, NG has become resistant to every antibiotic that has been used to treat it through all mechanisms of antimicrobial resistance (AMR) [4]. This has led to the threat of potentially untreatable gonorrhea, including a Canadian case resistant to ceftriaxone, further highlighting this concern [5]. Surveillance for antimicrobial resistance in gonorrhea is critical to monitor trends over time and to guide the development of treatment guidelines for gonorrhea [4]. Surveillance systems vary worldwide, but many focus exclusively on genital infections and employ the use of sentinel surveillance sites to access groups with a relatively high prevalence of NG in order to be cost effective [4].

Alberta (Canada) established a surveillance system in 2001 for AMR in NG. Initially, gonococcal cultures from genital and extragenital sites were obtained from STI clinics in the two main urban centers [6]. However, there was concern that the demographic and behavioral characteristics of individuals seeking testing at the STI clinics were different from those seeking testing outside of the STI clinics and, therefore, the resistance data may not be representative of the overall population infected with gonorrhea. In addition, individuals seeking testing outside of the STI clinics were mostly screened for NG using nucleic acid amplification tests (NAAT), thus precluding standard susceptibility testing. Beginning in 2015, in response to high rates of gonorrhea across the province, NAAT specimens from the northern half of the province were submitted to the National Microbiology Laboratory (NML) (Winnipeg, Manitoba, MB, Canada) for sequence typing with the primary goals of determining strain relatedness, identifying clusters within populations, and monitoring the spread of resistant clones, as well as transmission patterns within sexual networks [7].

The main objectives of our study were to: (1) assess the representativeness of our sentinel sites, (2) determine the antimicrobial resistance patterns of isolates, and (3) determine cluster characteristics through sequence-type analyses.

## 2. Materials and Methods 

Under Alberta’s Public Health Act, all cases of NG are reportable by all testing laboratories and testing clinicians to the provincial STI program. All clinical and behavioral data are submitted by the testing clinician on a Notification of STI Form and entered into a provincial database. NG was diagnosed by either nucleic acid amplification testing (NAAT) and/or culture. The three provincial STI clinics, located in Calgary, Edmonton, and Fort McMurray, were sentinel sites for NG antimicrobial susceptibility and routinely screened using both NAAT and culture. From culture-based specimens, the Provincial Laboratory (ProvLab) for Public Health conducted E-tests for susceptibility to cefixime, ceftriaxone, ciprofloxacin, azithromycin, penicillin, and tetracycline. Isolates demonstrating resistance, isolates with cefixime minimum inhibitory concentration (MIC) values of ≥0.06 μg/mL, susceptible isolates collected during the first 15 days of each month (beginning 2014), and NAAT specimens from the northern half of the province (beginning 2015) were submitted to the NML for sequence typing. 

Criteria for interpretation of resistance for NG E-test minimum inhibitory concentrations (MIC) values were based on the Clinical Laboratory Standards Institute (CLSI) standards (ciprofloxacin ≥ 1.0 μg/mL, penicillin ≥ 2.0 μg/mL, and tetracycline ≥ 2.0 μg/mL) [8]. Intermediate values for penicillin were 0.125–1.0 μg/mL, tetracycline were 0.5–1.0 μg/mL, and ciprofloxacin were 0.125–0.5 μg/mL; all values below the intermediate threshold were considered susceptible. Breakpoints for resistance to cefixime and ceftriaxone have not been defined by CLSI. However, isolates with an MIC of ≤0.25 μg/mL were considered susceptible. To understand characteristics associated with rising MIC values in the case-based analysis, cefixime MIC values were grouped into two categories: ≤0.016–0.03 μg/mL and ≥0.06 μg/mL. This upper range was chosen to capture differences between E-test results from the ProvLab and Agar dilution results from the NML, which can differ by 1 to 2 dilutions. CLSI does not provide interpretive criteria for azithromycin; an MIC value of ≥2.0 μg/mL is considered to have decreased susceptibility by the U.S. Center for Disease Control and Prevention’s (CDC) Gonococcal Isolate Surveillance Project (GISP) [9].

Sequence typing was performed using the *N. gonorrhoeae* Multi-Antigen Sequence Typing (NG-MAST) protocol and assigned using http://www.ng-mast.net/ as previously described [10,11]. Sequence groups (SGs) were created by finding other sequence typing (ST) that shared an identical *porB* or *tbpB* allele and that differed by ≤1% (≤5 base pairs difference for *porB* and ≤4 base pairs difference for *tbpB*) in the other allele. Each SG was named after the predominant ST of that group [12].

Data from the multiple sources were extracted for specimens collected between 2012 and 2016 and were merged by specimen number. One record per case in a reporting year was selected by sorting specimen-based data using the following algorithm of preferences, where applicable: (a) select culture-based record over NAAT; (b) select record with ST; (c) select record with maximum MIC considering the order of antibiotics as cefixime > ceftriaxone > azithromycin > ciprofloxacin > penicillin > tetracycline; and (d) select record in order of infection site as pharyngeal > genital > rectum. One NG-MAST ST per case was selected using the above mentioned algorithm, excluding step (a). 

Chi-square and Fisher’s exact tests were used for statistical analyses. In case of multiple small cell sizes, Monte Carlo estimation exact *p*-value was requested instead of direct exact *p*-value. The significance was set at *p*-value of <0.05. The analyses were performed using SAS^®^ 9.4 (SAS Institute Inc., Cary, NC, USA). Ethics approval was obtained from the University of Alberta’s Health Research Ethics Board.

## 3. Results

### 3.1. Culture Representativeness

Between 2012 and 2016, 13,132 NG cases were reported. Nearly one-quarter (22.0%; *n* = 2891) of cases had isolates available for susceptibility testing. Characteristics of cases were analyzed by test type to understand representativeness of culture-positive cases (Table 1). Significant differences between culture-positive cases and NAAT-only cases were found in all characteristics that were assessed. The proportion of cases tested by NAAT-only has increased in recent years. Cases that were culture-positive were more likely to be male, older, Caucasian, and reported same-sex partners. The Edmonton zone had the highest (56.2%; *n* = 1624) proportion of culture-positive cases among zones. The three sentinel sites (provincial STI clinics) reported 90.9% (*n* = 2628) of culture-positive cases, whereas 87.5% (*n* = 8964) of the NAAT-only cases were diagnosed by other providers (non-STI clinics).

### 3.2. Antimicrobial Resistance Patterns

**Figure 1:**
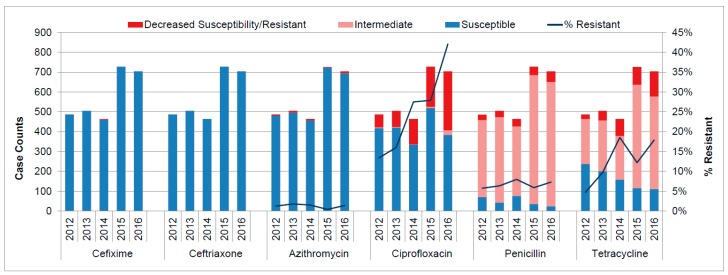
Antimicrobial resistance patterns for *Neisseria gonorrhoeae* tested in Alberta, 2012–2016.

Between 2012 and 2016, isolates from 2888 (of 2891) culture positive cases were tested for susceptibility to multiple antibiotics. All culture positive isolates were susceptible to ceftriaxone (Figure 1). The only findings of decreased susceptibility (0.5 ug/mL) to cefixime occurred in 2014 (four cases). All four cases reported opposite sex partners, three cases resided in Calgary, three cases were women, and all isolates were susceptible to ceftriaxone and azithromycin, resistant to ciprofloxacin, and had intermediate susceptibility to penicillin and tetracycline. 

Resistance to azithromycin (≥2 ug/mL) ranged between 0.4% (*n* = 3) and 1.8% (*n* = 9), without any trend in the study period. The proportion of cases resistant to ciprofloxacin increased from 13.4% (*n* = 65) in 2012 to 42.1% (*n* = 297) in 2016. Penicillin resistance remained between 5.8% (*n* = 28) and 8.0% (*n* = 37). Tetracycline resistance varied by reporting years. After a sharp increase to 18.5% (*n* = 86) in 2014, the proportion dropped to 12.2% (*n* = 89) in 2015 but climbed again to 17.9% (*n* = 126) in 2016.

### 3.3. Sequence Types

NG-MAST was completed on more than one-half (51.0%; *n* = 1473) of culture-positive cases. The proportion of culture-positive cases that were sequence typed increased from 18.5% (*n* = 90) in 2012 to two-thirds of culture-positive cases in 2014 (69.0%; *n* = 321), 2015 (69.1%; *n* = 504), and 2016 (60.7%; *n* = 427), due to the inclusion of all susceptible isolates from the first 15 days of each month. Prior to 2014, only selected isolates were sequence typed (those resistant to at least one antibiotic and/or had cefixime MIC ≥ 0.06 μg/mL). Beginning in 2015, NAAT specimens from the northern zone of the province were included in sequence typing, adding an additional 747 sequence typed cases. In total, 2220 cases had sequence type data available.

From 2012 to 2016, a total of 509 unique NG-MAST STs were identified, and 70.1% (*n* = 357) of these STs were newly identified in this time period. The most prevalent STs (>100 cases) were ST-7638 (*n* = 328), ST-5985 (*n* = 133), and ST-11299 (*n* = 116), which together represented 26.0% (*n* = 577) of all sequence typed cases. None of the three most prevalent STs were seen in 2012, and ST-11299 was not identified prior to 2014. Closely related sequence types were added to create SGs (Table 2).

SG-7638 was the most prevalent NG-MAST SG between 2012 and 2016. When compared to the other SGs, SG-7638 had the highest proportion of cases among women, First Nations, and Métis, opposite sex partners, from the North Zone, and from a non-STI testing provider, with the lowest proportion of isolates with resistance (Table 3). The second most prevalent SG was SG-5985. The majority of cases for SG-5985 were from men, Caucasians, and reported same sex partners. Cases were reported across all five years and from all five geographic areas. The majority of isolates were resistant to tetracycline. The third most prevalent SG was SG-11299. This SG was also predominantly found among men, Caucasians, and reported same sex partners. Cases were identified between 2013 and 2016 and reported in four of five geographic areas. The majority of isolates were resistant to ciprofloxacin, with some resistance to penicillin and tetracycline.

## 4. Discussion

The Alberta model for monitoring gonococcal antimicrobial susceptibilities, established in 2001, includes nonrandom, systematic sampling of male and female specimens from genital as well as extragenital sites at three sentinel sites. Such sampling minimizes selection bias since all clinic attendees undergoing physical assessments are offered screening using both NAAT and culture. Since the STI clinics maintain culture capacity, report 91% culture-positive cases in the province, and are in urban centers where samples are submitted to the Provincial Laboratory two times daily; these sites have served as practical locations to continue sentinel surveillance. However, surveillance programs aspire to collect samples from a representative section of the population to inform treatment guidelines. A comparison of the behavioral and demographic characteristics of individuals attending the STI clinics versus those seeking care elsewhere reveals significant differences between culture positive cases and NAAT-only cases, with culture positive cases more likely to be male, older, report same sex partners, and to be Caucasian, with the majority (56%) of culture-positive case from the Edmonton region. These differences provide further rationale for the inclusion of sequence testing to NAAT specimens to increase the representativeness of the surveillance system. Similar rises in rates of gonorrhea, especially among men, with rate increases in older age groups and marked geographic variation in rates have been observed in the U.S. [9]. In addition, similar to the U.S., our STI clinics diagnose a disproportionate number of NG infections among men [9]. In contrast to our surveillance program, however, the CDC’s GISP monitors antimicrobial susceptibility trends from male only urethral specimens collected at sentinel STI clinics in the U.S. [9]. Similar to our model, the United Kingdom’s Gonococcal Resistance to Antimicrobials Surveillance System (GRASP) collects specimens from genitourinary medicine clinic attendees (male and female) as well as extragenital and genital sites [13]. In the UK, gonorrhea tends to be concentrated among specific population groups, especially young adults, black Caribbean people, and men who have sex with men (MSM), but the majority of isolates in the GRASP sentinel system are from MSM (861/1284 = 67%), with 65% from those of White ethnicity and the highest proportion (42%) among those aged 20–24 [13]. Australia’s Gonococcal Surveillance Programme also tests isolates from males and females and genital and extragenital sites from public and private sector clinics, with 85% of isolates from males [14]. Geographical variation in populations affected by gonorrhea may in part reflect differences in the characteristics of the general population in those areas. 

Resistance has remained low to currently used drugs for treatment of NG with all isolates susceptible to ceftriaxone, and since 2015, all cases susceptible to cefixime. Resistance to azithromycin during the study time period ranged from 0.4–1.8%, well below the 5% threshold set by the World Health Organization (WHO), above which it is recommended that the agent not be routinely recommended for treatment [15]. Resistance to previously used drugs, however, remains high with penicillin resistance ranging from 5.8–8.0%, tetracycline 12.2–18.5%, and ciprofloxacin 13.4–42.1%, thus precluding the routine use of these antibiotics. 

NAATs have revolutionized testing for gonorrhea due to the improved sensitivity and specificity, ability to use non-invasive specimens, and stability during transportation of specimens, especially from remote settings, but the main disadvantage is routine testing for resistance from NAAT specimens is not currently routinely available in most places [16]. During the study period, 78% of specimens collected for NG testing used NAAT; this proportion has been increasing over time from 77% in 2012 to 81% in 2016. NG-MAST has the potential to identify strains which have been linked to reduced susceptibility to antibiotics and can be applied to culture as well as NAAT specimens. In an attempt to enhance the surveillance of specimens from northern Alberta where transportation delays preclude culture testing and rates were highest, NAAT specimens were collected for NG-MAST beginning in 2015. Analysis of the STs from both culture and NAAT showed 509 unique STs with 70% newly identified in this time period. Interestingly, SG-7638 was the most prevalent NG-MAST sequence group (SG) between 2012 and 2016, with the majority of cases occurring in 2015 and 2016 when our recent outbreak began. This strain has also been identified in the neighboring province of Saskatchewan [17]. SG-7638 had the highest proportion of cases among women, indigenous people, and those with opposite sex partners. The majority of cases were reported from the Edmonton and North zones, where the outbreak has the highest rates. Within this SG, 56.5% were isolates (vs. NAAT) and based on the NG-MAST profiles of SG-7638, the majority of the isolates were susceptible to the tested antimicrobials, indicating that the most common SG in our current outbreak is susceptible to our current treatment guidelines.

Among MSM, the most prevalent SGs were SG-5985 and SG-11299 with the majority among males and Caucasians reported in most years and from nearly all geographic areas. Although predominantly reported among cases with same sex partners, over one-quarter of cases reported opposite sex partners, suggesting that these SGs are circulating widely in the province and not restricted to one sub-population. ST-5985 was the most prevalent ST across Canada in 2016 while SG-5985 and SG-11299 have also been reported from neighboring Saskatchewan [17]. From these two SGs, nearly 90% of cases were available as isolates, with the majority of SG-5985 resistant to tetracycline and the majority of SG-11299 resistant to ciprofloxacin. This finding confirms that prevalent gonorrhea SGs circulating in Alberta have maintained resistance to previously used antimicrobial agents. 

Monitoring of ST in our province showed changes in the predominant types over time (See Section 3.3 above). None of the three most currently prevalent sequence groups were reported in 2012, suggesting an influx of different strains over time rather than the spread of a single clone of gonorrhea. The explanations for the waxing and waning of strains over time are unclear but most likely reflect spontaneous mutation of strains over time. Ongoing travel and mixing between communities within and between provinces as well as international travel raises the possibility that a resistant strain could enter and spread and highlights the need for continued surveillance.

Our study is not without limitations, including the geographical draw of the sampling framework which may result in STs that appear to be unique in a region. STs provide the most information when associated culture and susceptibility results can be linked to them. Since many unique STs were identified in our study that were not previously reported, the resistance profiles of the types obtained from NAAT specimens without accompanying culture specimens are not known. 

## 5. Conclusions

The Alberta model provides a consistent and systematic strategy for maintaining surveillance for antimicrobial resistance in gonorrhea using both culture and NAAT specimens. This information is crucial to informing provincial treatment guidelines. Future surveillance initiatives will look at enhancing ST from NAAT specimens by also testing these specimens for resistance genes to enhance the representativeness of samples and to include cases seen outside of STI clinics and in non-urban centers.

## Figures and Tables

**Table 1:** Characteristics of gonorrhea cases by test type, Alberta, 2012–2016; *N* = 13,132. NAAT = nucleic acid amplification testing.

Characteristics	Categories	Test Type		Total (*N* = 13,132)
		Culture (*n* = 2891)	NAAT-Only (*n* = 10,241)	
Year (*p* < 0.001)	2012	487 (16.8%)	1594 (15.6%)	2081 (15.8%)
	2013	506 (17.5%)	1491 (14.6%)	1997 (15.2%)
	2014	465 (16.1%)	1426 (13.9%)	1891 (14.4%)
	2015	729 (25.2%)	2682 (26.2%)	3411 (26.0%)
	2016	704 (24.4%)	3048 (29.7%)	3752 (28.6%)
Gender (*p* < 0.0001)	Male	2285 (79.0%)	5017 (49.0%)	7302 (55.6%)
	Female	606 (21.0%)	5224 (51.0%)	5830 (44.4%)
Age (in year) (*p* < 0.001)	0–14	10 (0.3%)	75 (0.7%)	85 (0.7%)
	15–19	256 (8.9%)	1723 (16.8%)	1979 (15.1%)
	20–24	648 (22.4%)	2818 (27.5%)	3466 (26.4%)
	25–29	780 (27.0%)	2226 (21.7%)	3006 (22.9%)
	30–34	520 (18.0%)	1532 (15.0%)	2052 (15.6%)
	35–39	242 (8.4%)	839 (8.2%)	1081 (8.2%)
	40 and older	435 (15.0%)	1028 (10.1%)	1463 (11.1%)
Ethnicity (*p* < 0.001)	First Nation	373 (12.9%)	3439 (33.6%)	3812 (29.0%)
	Inuit	2 (0.1%)	13 (0.1%)	15 (0.1%)
	Métis	148 (5.1%)	546 (5.3%)	694 (5.3%)
	Asian	206 (7.1%)	332 (3.3%)	538 (4.1%)
	Black	215 (7.4%)	448 (4.4%)	663 (5.0%)
	Caucasian	1761 (60.9%)	3699 (36.1%)	5460 (41.6%)
	Other	95 (3.3%)	155 (1.5%)	250 (1.9%)
	Unknown	91 (3.2%)	1609 (15.7%)	1700 (13.0%)
Sexual Partner (*p* < 0.001)	Opposite sex	1434 (49.6%)	7144 (69.8%)	8578 (65.3%)
	Same sex	1196 (41.4%)	801 (7.8%)	1997 (15.2%)
	Bisexual	187 (6.5%)	290 (2.8%)	477 (3.6%)
	Case < 12 years	7 (0.2%)	6 (0.1%)	13 (0.1%)
	Unknown	67 (2.3%)	2000 (19.5%)	2067 (15.8%)
Geographic Area (*p* < 0.001)	South	33 (1.1%)	246 (2.4%)	279 (2.1%)
	Calgary	1150 (39.8%)	1924 (18.8%)	3074 (23.4%)
	Central	15 (0.5%)	902 (8.8%)	917 (6.9%)
	Edmonton	1624 (56.2%)	4627 (45.2%)	6251 (47.6%)
	North	69 (2.4%)	2542 (24.8%)	2611 (20.0%)
Testing Agency (*p* < 0.001)	Calgary STI Clinic	1114 (38.5%)	281 (2.7%)	1395 (10.6%)
	Edmonton STI Clinic	1468 (50.8%)	878 (8.6%)	2346 (17.9%)
	Fort McMurray STI Clinic	46 (1.6%)	118 (1.2%)	164 (1.2%)
	Other Providers	263 (9.1%)	8964 (87.5%)	9227 (70.3%)

**Table 2:** *N. gonorrhoeae* Multi-Antigen Sequence Typing (NG-MAST) sequence groups (SGs) for prevalent sequence typing (ST) (Alberta, 2012–2016).

Sequence Group	Cases/Isolates (*n*)	Predominant ST (*n*)	Differ ≤1% for *porB* ST (*n*)	Differ ≤1% for *tbpB* ST (*n*)
SG-7638	367	ST-7638 (328)	ST-10815 (12), ST-12095 (1), ST-12863 (17), ST-13828 (1), ST-14537 (1), ST-14878 (1), ST-14984 (1)	ST-13826 (5)
SG-5985	145	ST-5985 (133)	ST-6968 (3), ST-11348 (1), ST-11471 (1), ST-11544 (1), ST-11841 (1), ST-14865 (3)	ST-10131 (2)
SG-11299	127	ST-11299 (116)	ST-8695 (4), ST-11837 (1), ST-12389 (5), ST-15200 (1)	--

**Table 3:** Case characteristics by NG-MAST group, Alberta, 2012–2016. STI = sexually transmitted infection; MIC = minimum inhibitory concentrations.

Characteristics	Categories	NG-MAST Group *n* (%)		
		SG-7638 (*n* = 367)	SG-5985 (*n* = 145)	SG-11299 (*n* = 127)
Gender	Female	147 (40.1%)	17 (11.7%)	15 (11.8%)
	Male	220 (59.9%)	128 (88.3%)	112 (88.2%)
Age (in years)	0–14	1 (0.3%)	1 (0.7%)	0
	15–19	35 (9.5%)	9 (6.2%)	6 (4.7%)
	20–24	93 (25.3%)	43 (29.7%)	25 (19.7%)
	25–29	103 (28.1%)	45 (31.0%)	31 (24.4%)
	30–34	59 (16.1%)	23 (15.9%)	22 (17.3%)
	35–39	38 (10.4%)	5 (3.4%)	20 (15.8%)
	40+	38 (10.4%)	19 (13.1%)	23 (18.1%)
Ethnicity	Asian	13 (3.5%)	8 (5.5%)	14 (11.0%)
	Black	19 (5.2%	6 (4.1%)	4 (3.2%)
	Caucasian	118 (32.2%)	109 (75.2%)	90 (70.9%)
	First Nation	134 (36.5%)	7 (4.8%)	4 (3.2%)
	Metis	44 (12.0%)	3 (2.1%)	5 (3.9%)
	Other	6 (1.6%)	5 (3.5%)	4 (3.2%)
	Unknown	33 (9.0%)	7 (4.8%)	6 (4.7%)
Sexual Partners	Opposite Sex	302 (82.3%)	42 (29.0%)	35 (27.6%)
	Same Sex	16 (4.3%)	93 (64.1%)	69 (54.3%)
	Bisexual	15 (4.1%)	5 (3.4%)	17 (13.4%)
	Case < 12 years	1 (0.3%)	1 (0.7%)	0
	Unknown	33 (9.0%)	4 (2.8%)	6 (4.7%)
Year	2012	0	3 (2.1%)	0
	2013	1 (0.3%)	22 (15.2%)	4 (3.2%)
	2014	8 (2.2%)	47 (32.4%)	22 (17.3%)
	2015	229 (62.4%)	45 (31.0%)	52 (40.9%)
	2016	129 (35.1%)	28 (19.3%)	49 (38.6%)
Geographic Area	South	2 (0.5%)	1 (0.7%)	1 (0.8%)
	Calgary	29 (8.0%)	82 (56.5%)	48 (37.8%)
	Central	2 (0.5%)	1 (0.7%)	0
	Edmonton	153 (41.7%)	42 (29.0%)	64 (50.4%)
	North	181 (49.3%)	19 (13.1%)	14 (11.0%)
Testing Agency	Calgary STI Clinic	28 (7.6%)	81 (55.9%)	45 (35.4%)
	Edmonton STI Clinic	131 (35.7%)	36 (24.8%)	60 (47.2%)
	Fort McMurray STI Clinic	13 (3.5%)	5 (3.4%)	1 (0.8%)
	Other providers	195 (53.1%)	23 (15.9%)	21 (16.5%)
Antibiotic Resistance or Decreased Susceptibility (MIC Value; µg/mL) ^1^	Culture-positive Isolates	192 (52.3%)	131 (90.3%)	114 (89.8%)
	Cefixime (>0.25)	0	0	0
	Ceftriaxone (>0.25)	0	0	0
	Azithromycin (>2)	1 (0.5%)	0	0
	Ciprofloxacin (≥1)	6 (3.1%)	1 (0.8%)	108 (94.7%)
	Penicillin (≥2.0)	0	0	21 (18.4%)
	Tetracycline (≥2.0)	3 (1.6%)	128 (97.7%)	8 (7.0%)

^1^ Proportions calculated based on number of culture-positive isolates.
